# Characterization of Tetratricopeptide Repeat-Containing Proteins Critical for Cilia Formation and Function

**DOI:** 10.1371/journal.pone.0124378

**Published:** 2015-04-10

**Authors:** Yanan Xu, Jingli Cao, Shan Huang, Di Feng, Wei Zhang, Xueliang Zhu, Xiumin Yan

**Affiliations:** 1 State Key Laboratory of Cell Biology, Institute of Biochemistry and Cell Biology, Shanghai Institutes for Biological Sciences, Chinese Academy of Sciences, 320 Yueyang Road, Shanghai, China; 2 School of Life Science and Technology, ShanghaiTech University, 100 Haike Road, Shanghai, China; Institute of Molecular and Cell Biology, SINGAPORE

## Abstract

Cilia formation and function require a special set of trafficking machinery termed intraflagellar transport (IFT), consisting mainly of protein complexes IFT-A, IFT-B, BBSome, and microtubule-dependent molecular motors. Tetratricopeptide repeat-containing (TTC) proteins are widely involved in protein complex formation. Nine of them are known to serve as components of the IFT or BBSome complexes. How many TTC proteins are cilia-related and how they function, however, remain unclear. Here we show that twenty TTC genes were upregulated by at least 2-fold during the differentiation of cultured mouse tracheal epithelial cells (MTECs) into multiciliated cells. Our systematic screen in zebrafish identified four novel TTC genes, *ttc4*, *-9c*, *-36*, and *-39c*, that are critical for cilia formation and motility. Accordingly, their zebrafish morphants displayed typical ciliopathy-related phenotypes, including curved body, abnormal otolith, hydrocephalus, and defective left-right patterning. The morphants of *ttc4* and *ttc25*, a known cilia-related gene, additionally showed pronephric cyst formation. Immunoprecipitation indicated associations of TTC4, -9c, -25, -36, and -39c with components or entire complexes of IFT-A, IFT-B, or BBSome, implying their participations in IFT or IFT-related activities. Our results provide a global view for the relationship between TTC proteins and cilia.

## Introduction

Cilia are evolutionarily conserved, microtubule-based, hair-like organelles present in most animal cells. They play important roles in cell movement, environment sensing, and signal transduction [1–3]. Defects in cilia assembly and function result in severe disorders called ciliopathies, including infertility, retinal degeneration, hydrocephalus, polycystic kidney, and *situs inversus* [2,4,5]. Therefore, it is important to elucidate the mechanisms underlying ciliogenesis and ciliary functions for the understanding and the treatment of ciliopathies.

The assembly, maintenance and functions of cilia depend on the intraflagellar transport (IFT), the bidirectional trafficking of vesicles and proteins along the ciliary axoneme [6]. Previous works have revealed that the IFT is mediated by motor protein complexes, cytoplasmic dynein and kinesins, via IFT particles that serve as cargo acceptors [3,7]. IFT particles can be further classified into two subcomplexes termed IFT-A (containing IFT144, -140, -139, -122, -121, and -43) and-B (containing IFT172, -88, -81, -80, -74, -70, -57, -54, -52, -46, -27, -25, -22, and -20) [3,8]. Their assembly at the cilia base and turnaround at the cilia tip requires another protein complex, BBSome [9–11]. BBSome is composed of seven proteins (BBS1, -2, -4, -5, -7, -8, and -9) [3,12]. Mutations in these components cause Bardet-Biedl syndrome (BBS), an autosomal recessive disorder with polydactyly, kidney cysts, nephropathy, and obesity [3,13].

The tetratricopeptide repeat (TPR) domain is a structural domain that mediates protein-protein interactions important for multiprotein complex formation. It usually consists of 3–16 TPRs, each of which is a motif of 34 amino acids [14–16]. Crystallographic analysis on phosphatase 5 shows that the TPR motif forms a pair of antiparallel α-helices and multiple TPRs are arranged in a parallel manner to form a right-handed superhelix [17]. The TPR-containing (TTC) proteins widely exist from bacteria to humans and are involved in many important biological processes, such as intracellular transport, vesicle fusion, protein folding, cell cycle, and transcriptional regulation [16]. Interestingly, TPR domains are found in all the IFT-A subunits except IFT122 and IFT43, in IFT172, IFT88, and IFT70 of the IFT-B complex, and in BBS4 and BBS8 of BBSome [18–20]. TTC26 has recently been shown as a component of IFT-B complex [21], whereas *Xenopus* TTC25 is found to be important for ciliogenesis and the signal transduction of sonic hedgehog pathway, a cilium-dependent signaling pathway in vertebrate [22]. Whether there are novel TTC proteins involved in cilia formation and function, however, is not clear.

In this study, we used cDNA microarray to screen for TTC proteins up-regulated during multiciliogenesis of cultured mouse tracheal epithelial cells (MTECs) [23], and characterized their roles in cilia formation and functions during the embryonic development of zebrafish. In addition to known cilia-related TTC proteins, we identified four novel TTC proteins critical for cilia formation and functions. Furthermore, our results suggest that all these TTC proteins are involved in IFT.

## Materials and Methods

### Ethics Statement

All experimental operations involving zebrafish were approved by the Institutional Animal Care and Use Committee of Shanghai Institute of Biochemistry and Cell Biology (Animal Use Protocol number: IBCB-ZF012).

### Plasmids and mRNAs

The full-length cDNAs of human *TTC4* (NM_004623), human *TTC9c* (NM_173810), human *TTC25* (NM_031421), mouse *TTC36* (NM_138951) and mouse *TTC39c* (NM_028341) were amplified by RT-PCR from HEK293T cells or mouse testis and cloned into pcDNA3.1-FLAG to express FLAG-tagged proteins in mammalian cells. For antibody productions, the full-length cDNAs were subcloned into pET28a to express polyhistidine (His)-tagged TTC proteins in *E*. *coli* to serve as antigens. Full-length cDNAs were also subcloned into pGEX-4T-1 to express GST-tagged TTC proteins in *E*. *coli* for antigen competition experiment. For GST pull-down assays, the full-length cDNA of mouse *BBS7* (NM_027810) was amplified by RT-PCR from mouse testis and cloned into pGEX-4T-1.

For the rescue experiments, the full-length cDNAs of zebrafish *ttc4* (NM_001002122), *ttc9c* (NM_200265), *ttc36* (NM_001007388), and *ttc39c* (NM_001020568) were amplified by RT-PCR from 48-hpf zebrafish embryos and rendered morpholino oligonucleotide (MO)-resistant by introducing five mismatched synonym nucleotides in the MO-targeting sequences. The cDNAs were subcloned into pCS2^+^ and used for the syntheses of capped mRNAs with mMESSAGE mMACHINE SP6 kit (Ambion, AM1340).

The GFP reporters for MO efficiency analyses were constructed in pCS2^+^. An approximately 200-bp fragment of each zebrafish TTC cDNA containing the MO targeting sequence was cloned both upstream of and in frame with the coding sequence of EGFP. The mRNAs for the GFP reporters were *in-vitro* transcribed with mMESSAGE mMACHINE SP6 kit (Ambion, AM1340).

Primers used are listed in S1 Table. All the constructs were verified by sequencing.

### Cell culture and transfection

HEK293T cells (ATCC, CRL-3216^TM^) were cultured in DMEM supplemented with 10% fetal bovine serum, 0.3 mg/ml L-glutamine, 100 units/ml penicillin, and 100 units/ml streptomycin at 37°C in 5% CO_2_. The cells were transfected by the calcium phosphate method. MTECs were isolated and cultured as described before [24]. Briefly, MTECs were isolated from 8-week old C57BL/6J mice and were freshly seeded onto transwells with 0.4-μm polyester membrane (Corning, NY 14831). 10 μM DAPT (N-[N-(3,5-Difluorophenacetyl-L-alanyl)]-(S)-phenylglycine t-butyl ester) (Sigma), an inhibitor of the Notch signaling pathway, was added to increase the percentage of multiciliated cells [25].

### Morpholino oligonucleotides and microinjection

The control MO (*ctrl*-MO) is the Morpholino Standard Control from Gene Tools. The antisense MOs were designed and ordered from Gene Tools. Their sequences are as follows, with the underlined bases complementary to the initiation codon ATG:

*zttc4*-MO, 5’-CGTTGGTGCAGCCATGTTATCTCCT-3’;

*zttc5*-MO, 5’-CTCCATCATTGTCAACCTCGGCCAT-3’;

*zttc9c*-MO, 5’-GAGGATCTGCAACCTTTTCGTCCAT-3’;

*zttc12*-MO, 5’-AGACATGGTTGAAACAGAGTTCATA-3’;

*zttc16*-MO, 5’-GGATTCATATTGGCTTAAAGGTTAT-3’;

*zttc18*-MO, 5’-TCCCGAACTCTCTGTTATTCTCCAT-3’;

*zttc25*-MO, 5’-CTTGTCCTTCCTCGTTATCGAACAT-3’;

*zttc29*-MO, 5’-TCGAAGACATGCTGCTGAGTAAGTT-3’;

*zttc36*-MO, 5’-CTGCTCTGTCGTGTGCTGATGCCAT-3’;

*zttc39a*-MO, 5’-GTGCATTTTTCCCAGTGGACATGGT-3’;

*zttc39c*-MO, 5’-GGCCCGCCATCCTTTCTCCTTTTCT-3’;

MOs were dissolved in nuclease-free water and injected into the yolk of one-cell-stage zebrafish embryos by using Narishige IM300 microinjector as described before [24].

In the initial screen, a dosage of 8 ng per embryo was used [26–28]. As the morphants of *ttc2*5, *-4*, *-9c*, *-36*, and *-39c*, but not of the rest, displayed some embryonic death, we further examined the dose-dependent effects (S1 Fig) and optimized the dosage for each of the MOs to minimize the death effect. Accordingly, all the presented experimental data were achieved with the following dosages: 4 ng per embryo for the MO of *ttc25*, *-9c*, *-36*, or *-39c*; 6 ng per embryo for the MO of *ttc4*; 8 ng per embryo for the MOs of the remaining TTC genes; 6 or 8 ng per embryo for the control MO to match the maximal dosage of TTC MOs in the experiments.

To validate the knockdown efficiency, 100 pg of *in-vitro* transcribed GFP-reporter mRNA were coinjected with either the corresponding MO or *ctrl*-MO per zebrafish embryo. The GFP autofluorescent and bright-field images of the embryos were acquired at 12 hpf using a SZX16 stereomicroscope (Olympus, Tokyo, Japan).

In the rescue experiments, 400 pg of *in-vitro* transcribed mRNA per embryo were coinjected with MO. The phenotypes were assayed at 72 hpf.

### Antibodies

Rabbit polyclonal antibodies against TTC4 (NP_004614), TTC9c (NP_776171), TTC25 (NP_113609), TTC36 (NP_620401), and TTC39c (NP_082617) were raised in Shanghai Immune Biotech. Monoclonal acetylated-tubulin antibody (T6793) was from Sigma. Primary antibodies against IFT144 (13647-1-AP), IFT140 (17460-1-AP), IFT88 (13967-1-AP), IFT57 (11083-1-AP), IFT52 (17534-1-AP), and GAPDH (10494-1-AP) were from Proteintech (Chicago, IL). Primary antibodies against IFT139 (HPA035495) and IFT46 (HPA037909) were form Sigma. Secondary antibodies conjugated with Alexa Fluor 488, -546, or HRP were from Lifes.

### Fluorescence microscopy

MTECs were fixed and stained as described previously [29]. Briefly, MTECs grown on transwells were fixed with 4% paraformaldehyde in PBS for 10 min at room temperature. After fixation, cells were permeabilized with 0.5% Triton X-100 in PBS for 15 min and blocked with 1% BSA in PBS for 1 h. The incubations of primary and secondary antibodies were carried out at room temperature for 2 h and 1h, respectively. To validate the specificity of the homemade TTC antibodies, antigen competition experiments were performed as described [30,31]. Briefly, each antibody was pre-incubated with 15 μg of GST or GST-tagged antigen for 2 h at 4°C. After a 10-min full spin in a benchtop centrifuge, the supernatant was used for immunostaining.

Zebrafish embryos were manually dechorionated and fixed with 4% paraformaldehyde in PBS at room temperature for 4 h. After dehydration and rehydration, embryos were incubated with the antibody against acetylated tubulin (1:1,000) overnight at 4°C. After washing with 0.5% Triton X-100 in PBS, the embryos were incubated with Alexa Fluor-488-conjugated secondary antibody (1:1,000) for 2 h. After extensive washing, the embryos were embedded in 1% low-melting-point agarose.

Images were taken by using a Leica TCS SP5 MP confocal microscope. Optical sections were captured at 0.5-μm intervals and z-stack images were obtained by maximum intensity projections. Positions of the KV were determined through brightfield view of the 7-somite-stage zebrafish embryos prior to the acquisition of the fluorescent images.

### High-speed video microscopy

High-speed video microscopy for cilia motility was performed as described with some modifications [32]. Briefly, zebrafish embryos were treated with N-phenylthiourea (PTU, Sigma) at 24 hpf to inhibit pigmentation [33]. At 60 hpf, zebrafish were treated with 40 mmol/l BDM (2,3-butanedione monoxime, Sigma) and then embedded in 1% low melting agarose (Sigma) and the images of cilia beating were captured at 200 frames per second (fps) with IX71 inverted microscopy (Olympus) equipped with a 63 × oil-immersion objective lens and a Neo sCMOS camera (Andor). For each examined gene, cilia motilities of 10 randomly picked morphants were recorded.

### In situ hybridization and histology

*In situ* hybridization was performed as described before [24]. The *cmlc2* RNA probe was labeled using the DIG RNA labeling kit (Roche) and the probe concentration used was 2 μg/ml. Hybridization was performed overnight at 65°C.

For hematoxylin-eosin staining, embryos at 72 hours post fertilization (hpf) were fixed with 4% paraformaldehyde in PBS at room temperature for 4 h and embedded in paraffin after a gradual dehydration in ethanol. The embryos were cross-sectioned at a thickness of 5 μm using a rotary microtome (Leica RM 2235). Slides were then stained with hematoxylin and eosin.

### Microarray hybridization and data analysis

To screen for genes upregulated during ciliogenesis, total RNAs of MTECs samples from ALI d0 to d5 were isolated and hybridized to a GeneChip Mouse Genome 430 2.0 array (Affymetrix) through a contracted service (CapitalBio). About 900 genes showed increased hybridization signals by at least 2 folds during multiciliation. The results for selected genes are shown in S2 Table.

### Co-immunoprecipitation and GST pull-down

HEK293T cells transiently expressing FLAG-tagged proteins were lysed in the lysis buffer [20 mM Tris-Cl (pH 7.5), 100 mM KCl, 1% NP-40, 1 mM EDTA, 10% glycerol, 10 mM sodium pyrophosphate, 1 mM PMSF, 3 mM DTT, and protease inhibitor cocktail (Calbiochem)]. 3 ml of cell lysates from ~ 2 × 10^7^ cells were incubated with 3 ml of testis lysates from six 8-week mice for 2 h at 4°C. After the incubation, co-IP experiments were carried out with anti-FLAG resin (Sigma) as described previously [34].

GST-pull down experiments were performed as described before [34]. Briefly, bacterially expressed GST-BBS7 bound to glutathione-agarose beads (Sigma) were incubated respectively with bacterial lysate containing His-tagged luciferase, TTC25, TTC4, TTC9c, TTC36, or TTC39c for 2 h at 4°C. The beads were rinsed with wash buffer [20 mM Tris-Cl (pH 7.5), 150 mM KCl, 0.5% NP-40, 1 mM EDTA, 10% glycerol, 10 mM sodium pyrophosphate, 1 mM PMSF, and protease inhibitor cocktail (Calbiochem)] for three times. The samples were then analyzed by western blotting.

### Quantitative PCR

2 μg of total RNA was used for each reverse transcription reaction using the Superscript III First Strand Synthesis System with oligo-dT primers (Lifes). Quantitative PCR (qPCR) was performed by using an Applied Biosystems 7500 HT Sequence Detection System with the Power SYBR Green PCR Master Mix Kit (Applied Biosystems, Foster City, CA). GAPDH served as the control.

### Quantification and statistical analysis

Cilia length was measured using Image J (Media Cybernetics). Statistical results, obtained from three independent experiments in a blind manner, were presented as mean values and standard deviations.

Cilia beat frequencies (CBFs) were calculated by playing the movies frame by frame for five complete beat cycles. For each group of zebrafish morphants, 40 cilia from 4 randomly picked embryos were used for statistical analysis. Only cilia that were clearly traceable in the time-lapse movies were used for the analysis.

## Results

### Multiple TTC genes are upregulated during MTEC differentiation

**Fig 1:**
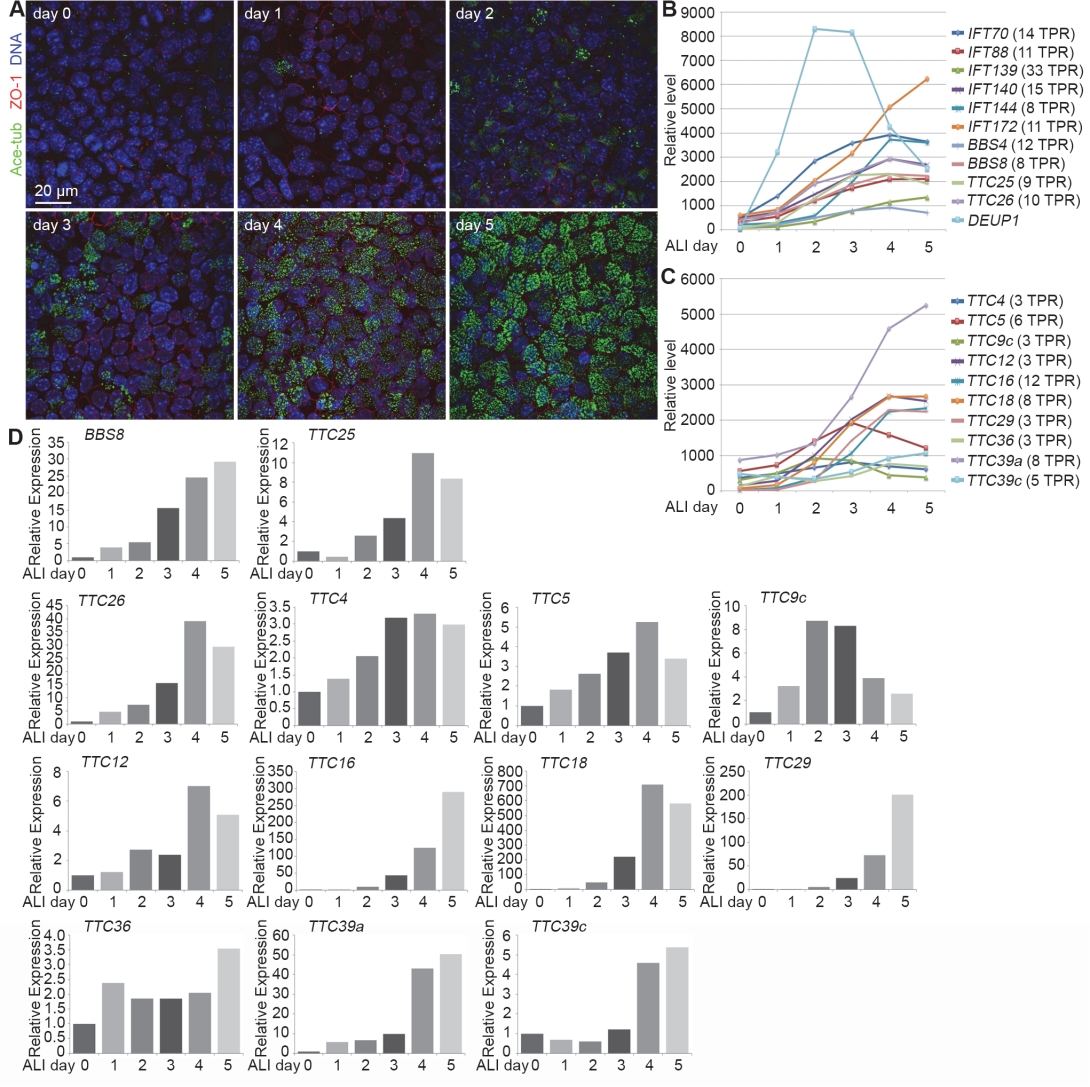
The expression profiles of TTC genes upregulated during MTEC differentiation into multiciliated cells. (A) Typical differentiation progression of MTECs cultured *in vitro*. MTECs were isolated from mice of 8-week old and were induced to differentiate into multiciliated cells by culturing at an air-liquid interface (ALI) for the indicated time. Approximately 90% of MTECs were multiciliated at ALI day 5. Acetylated tubulin (Ace-tub) was used to mark cilia. ZO-1 labels the tight junctions. (B) The gene expression profiles of known cilia-related TPR-containing proteins following the MTEC differentiation. The data were from cDNA microarray analyses on MTECs samples from ALI d0 to ALI d5. The gene expression pattern of Deup1, a protein critical for centriole amplification, is also included for comparison. (C) The expression profiles of ten novel TTC genes. (D) Expression profiles of the indicated genes, based on quantitative PCR (qPCR) results. One set of typical results of two independent experiments is presented (Please see S2 Fig for the second set of results). The mRNA levels at ALI d0 were set as 1.

Following the establishment of an air-liquid interface (ALI), MTECs cultured *in vitro* simultaneously generate hundreds of centrioles, which serve as the basal bodies of cilia, followed by the formation of dense motile cilia (Fig 1A) [23,29]. Accordingly, genes important for centriole biogenesis and cilia formation are upregulated. In our previous studies, we have demonstrated that during MTEC differentiation the centriole amplification peaks at ALI day 3 (d3) and multiciliation reaches plateau at around ALI d5 [24,29]. In the presence of DAPT, approximate 90% of the cells became multiciliated at ALI d5 (Fig 1A) [24,29]. Therefore, we performed cDNA microarray analyses with MTECs samples from ALI d0 to d5 to identify cilia-related genes.

Our microarray analyses identified approximately 900 upregulated genes, i.e., genes whose microarray signals increased by at least 2-fold, during the multiciliation of MTECs. As expected, the mRNA level of genes important for centriole biogenesis, such as DEUP1 (Fig 1B and S2 Table) [29], peaked at ALI d2-d3, whereas genes related to cilia elongation, such as the components of BBSome and IFT complexes, reached their highest expression levels from ALI d4-d5 (Fig 1B and S2 Table). Interestingly, twenty of the upregulated genes encoded TTC proteins. Compared to ALI d0, their microarray signals increased by 2 to 121 folds in later ALI days (Fig 1B and 1C and S2 Table). Half of them, including the TTC components of IFT-A,-B, and BBSomes and TTC25 and TTC26, are known to function in cilia (Fig 1B) [21,22,35]. The remaining ten, however, did not have documented cilia-related functions (Fig 1C).

We then validated the microarray results with qPCR. Similar to the known cilia-related genes, *BBS8*, *TTC25*, and *TTC*26, the mRNA levels of all the ten novel TTC genes increased during ALI d0 and d5 (Fig 1D and S2 Fig), correlated with ciliogenesis and cilia function.

### Zebrafish morphants of *ttc4*, *-9c*, *-36*, and *-39c* display abnormal body curvatures

**Fig 2:**
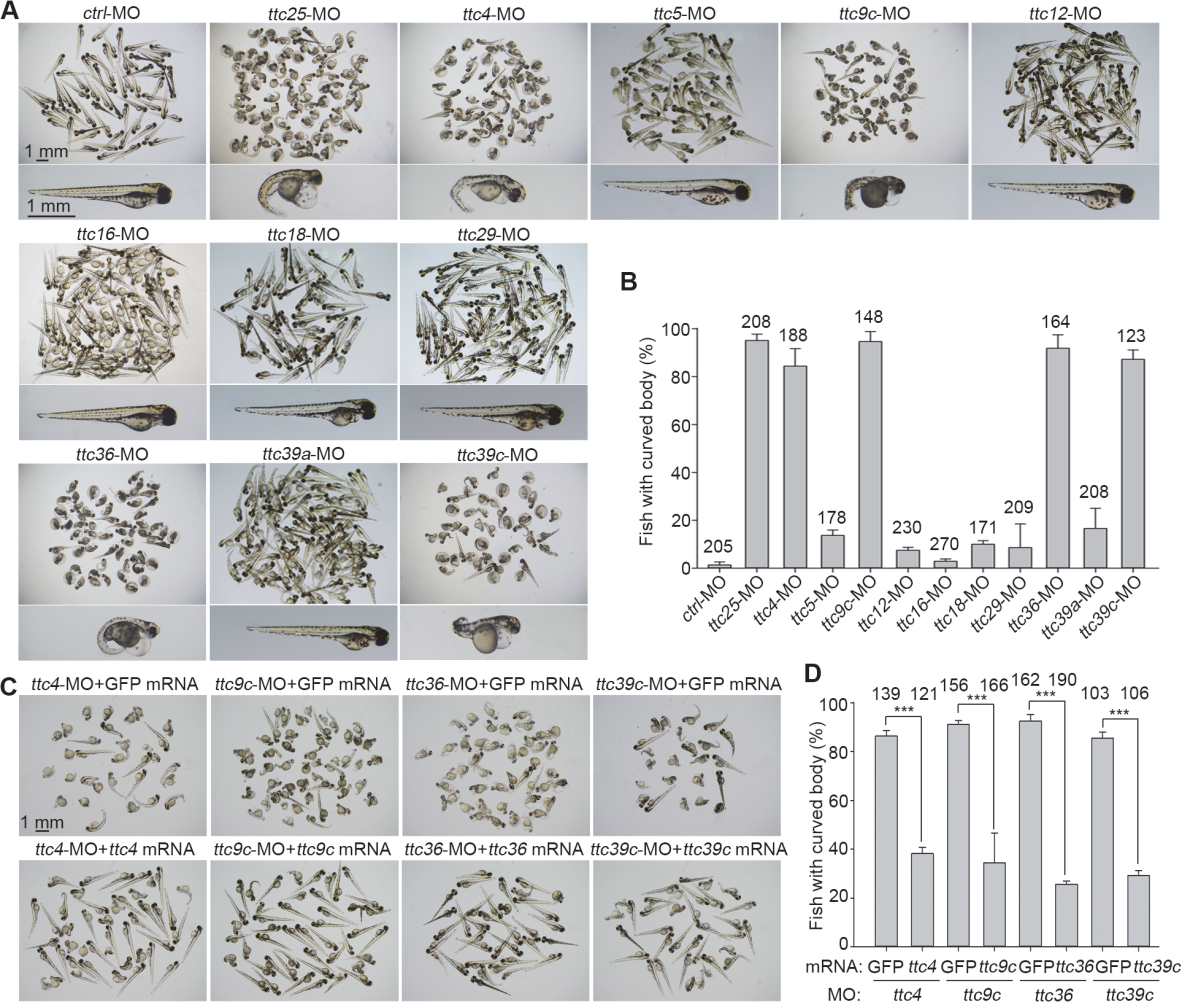
Screen for cilia-related TTC genes in zebrafish. (A) The morphologies of zebrafish morphants at 72 hpf. Note that the morphants of *ttc4*, *-9c*, *-25*, *-36*, and *-39c* displayed severe body curving. (B) Quantification results for the curved body phenotype. Three independent experiments were performed. (C) and (D) Rescue experiments for the *ttc* morphants. The indicated MO was coinjected with either the corresponding MO-resistant mRNA or GFP mRNA into zebrafish embryos. The body curvature phenotype was assayed at 72 hpf. The numbers of embryos counted are listed over the histograms. Statistical results are from three independent experiments. Student’s *t*-test, ***P < 0.001. Error bars represent s.d.

Zebrafish (*Danio rerio*) is an excellent vertebrate model animal because cilia-associated defects result in ciliopathy-related phenotypes, such as curved bodies, abnormal otoliths (ear stones), kidney cysts, hydrocephalus, and left-right patterning defects, during the embryonic development [24,32,36]. To determine whether the ten novel TTC genes had cilia-related functions, we repressed their protein expressions in zebrafish embryos with antisense MOs. Compared to the control fish, the *ttc25* morphants exhibited severe curved body phenotype at 72 hpf (95.1% vs. 1.3%) as expected (Fig 2A). In contrast to the morphants of *ttc5*, *-12*, *-16*, *-18*, *-29*, and *-39a*, whose abnormality incidence was below 17%, more than 84% of the morphants of *ttc4*, *-9c*, *-36*, and *-39c* developed curved bodies [36,37] (Fig 2A and 2B). To validate the efficiency and specificity of the MOs against *ttc4*, *-9c*, *-36*, and *-39c*, both GFP-reporter assays and rescue experiments were carried out. In contrast to the control MO, all the *ttc* MOs silenced their corresponding GFP-reporters (S3 Fig). More importantly, the body curvature phenotypes of the morphants were rescued upon coinjection of the corresponding MO-resistant mRNA of zebrafish *ttc4*, *-9*c, *-36*, or *-39c*, but not of the mRNA of EGFP (Fig 2C and 2D). These results suggest that *ttc4*, *-9c*, *-36*, and *-39c* could be cilia-related genes. We thus performed further analyses on them.

### Zebrafish morphants of *ttc4*, *-9c*, *-36*, and *-39c* develop multiple ciliopathy-related phenotypes

**Fig 3:**
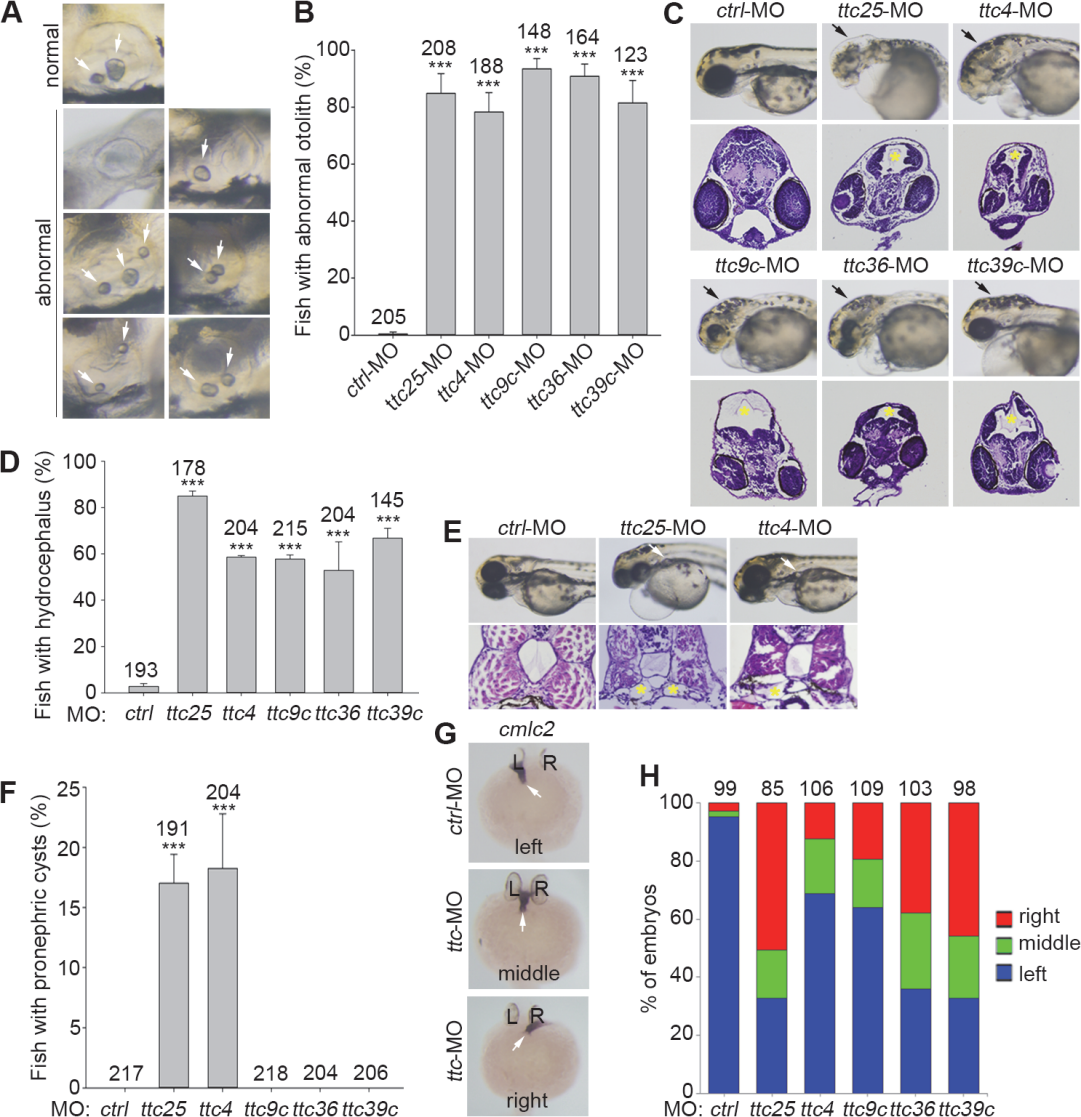
Additional phenotypes of the zebrafish morphants of *ttc4*, *-9c*, *-25*, *-36*, and *-39c*. (A) Typical otolith morphologies at 72 hpf. The otic vesicle normally contains two otoliths as shown. Otoliths that show differences in number, size and/or position were considered as abnormal. (B) Quantification results for abnormal otoliths in the indicated morphants. (C) and (D) Hydrocephalus formation (arrows in the bright-field images and asterisks in the histochemical sections) in the indicated morphants at 72 hpf. (E) and (F) The *ttc25* and *ttc4* morphants tended to develop pronephric cysts (arrows in the bright-field images and asterisks in the histochemical sections) at 72 hpf. (G) and (H) Left-right asymmetry patterning was disturbed in the indicated *ttc* morphants at 30 hpf. The *cmlc2* probe was used to label the heart tube (arrows) in whole-mount *in situ* hybridization. All the quantification results were based on three independent experiments. Student’s *t*-test, ***P < 0.001. Error bars represent s.d.

Next, we analyzed other ciliopathy-related phenotypes. There are two otoliths of unequal sizes in the inner ear of zebrafish (Fig 3A) [38]. Since proper otolith formation requires cilia motility in the inner ear, defects in the inner ear motile cilia often lead to abnormal otoliths, which are fused, mispositioned, or of wrong sizes or numbers [39]. We found that, similar to the *ttc25* morphants, more than 75% of the morphants of *ttc4*, -*9c*, -*36*, and -*39c* developed abnormal otoliths at 72 hpf, whereas in the control morphants the incidence was only 0.4% (Fig 3A and 3B).

We found that more than 52% of the *ttc* (including *ttc25*) morphants at 72 hpf exhibited bulged head phenotype resembling hydrocephalus, compared to the value (2.7%) for the control animals (Fig 3C and 3D). To confirm that the phenotype was indeed due to hydrocephalus, we performed histological analyses on brain cross-sections of the affected *ttc* morphants and confirmed that such morphants indeed contained dilated ventricles of varying severities compared to control (Fig 3C).

The incidences of pronephric cysts were relatively low. Obvious pronephric cysts were observed in 17.0% of *ttc25* and 18.3% of *ttc4* morphants at 72 hpf, but none in the other three *ttc* morphants and control animals (Fig 3E and 3F).

Last, we examined the left-right patterning of the internal organs. When the heart tube was labeled through *in situ* hybridization of *cardiac myosin light chain 2* (*cmlc2*) at 30 hpf [24,40], its leftward orientation was observed in 95.4% of control morphants (Fig 3G and 3H). By contrast, the *ttc* morphants exhibited varying extent of defects in the left-right asymmetry. More than 64% of the morphants of *ttc25*, *-36*, and *-39c* showed misoriented heart tubes toward either the right or the middle side, indicating a randomized heart tube orientation (Fig 3G and 3H). The morphants of *ttc4* and *ttc9c* showed milder defects: 31.1% and 35.8% of them had misoriented heart tubes, respectively (Fig 3G and 3H).

### *ttc4*, *-9c*, *-36*, and *-39c* are critical for cilia formation and motility

**Fig 4:**
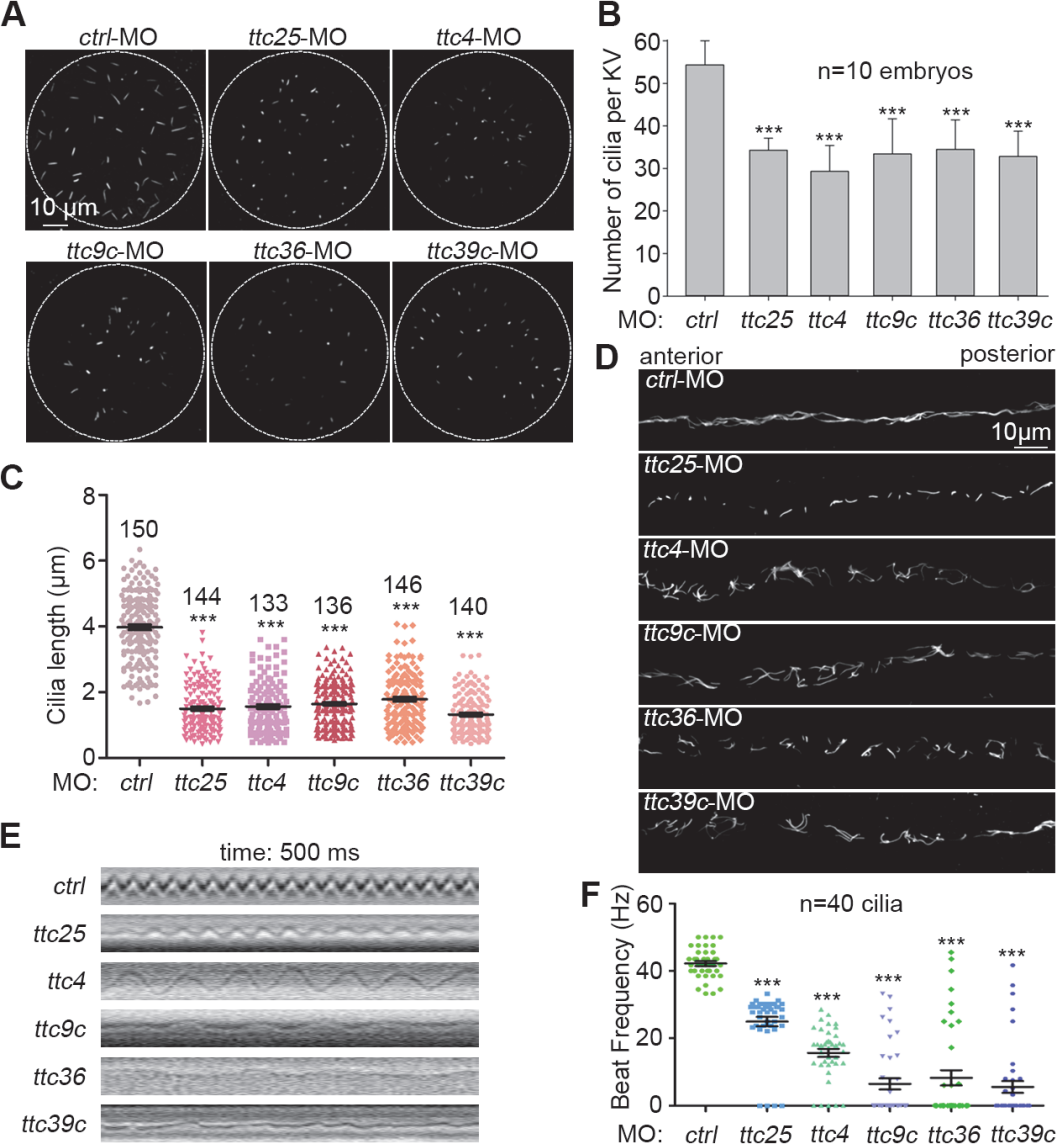
Examinations on cilia in the Kupffer’s vesicle and pronephric duct. (A-C) Both cilia number and cilia length in the Kupffer’s vesicle (KV) at the 7-somite stage were reduced in the indicated *ttc* morphants. Cilia were labeled using antibody against acetylated tubulin. The quantification results were based on three independent experiments. Student’s *t*-test, ***P < 0.001. Error bars represent s.d. (D) Typical morphology of cilia in pronephric ducts at 24 hpf. (E) and (F) Cilia motility in the pronephric duct at 60 hpf. The kymographs showed trajectories of the cilia marked with red line in S1, S2, S3, S4, S5 and S6 Videos. Cilia beat frequencies (CBFs) were measured at 60 hpf (40 cilia from 4 morphants for each gene). Student’s *t*-test, ***P < 0.001. Error bars represent s.e.m.

We then investigated whether the ciliopathy-related defects of the zebrafish morphants were indeed attributed to cilia defects. Kupffer’s vesicle (KV), a monociliated organ functionally equivalent to mouse embryonic node, is essential for establishment of the left-right asymmetry during early stages of embryonic development [32,41]. We thus examined KV cilia in embryos at the 7-somite stage and found that both the number and length of the KV cilia were significantly reduced in the *ttc* morphants (Fig 4A–4C). Furthermore, when the motile cilia in the pronephric duct were examined for zebrafish embryos at 24 hpf [24,32], we found that they were apparently disorganized in the *ttc* morphants as compared to the control ones (Fig 4D), suggesting defects in cilia motility. Quantification for the cilia number and length, however, was difficult due to intertwining of the cilia.

We next examined cilia motility in 60-hpf pronephric ducts by using high-speed video microscopy. In the control morphants, cilia beat in a rhythmic sinusoidal pattern (Fig 4E and S1 Video) [42]. The cilia beat was markedly impaired in the *ttc* morphants and no longer synchronized (Fig 4E and S2, S3, S4, S5 and S6 Videos) [43]. Strikingly, many cilia were paralyzed in the *ttc9c*, *ttc36*, and *ttc39c* morphants (Fig 4E and S4, S5 and S6 Videos). Quantification indicated that, while the average CBF was 42.2 ± 0.7 Hz in the control morphants, it became 25.0 ± 1.4Hz for *ttc25*, 15.7 ± 1.2 Hz for *ttc4*, 6.5 ± 1.7 Hz for *ttc9c*, 8.2 ± 2.2 Hz for *ttc36*, and 5.6 ± 1.8 Hz for *ttc39c* morphants, respectively (Fig 4F).

### TTC4, -9c, -25, and -36 localize to multicilia in MTECs

**Fig 5:**
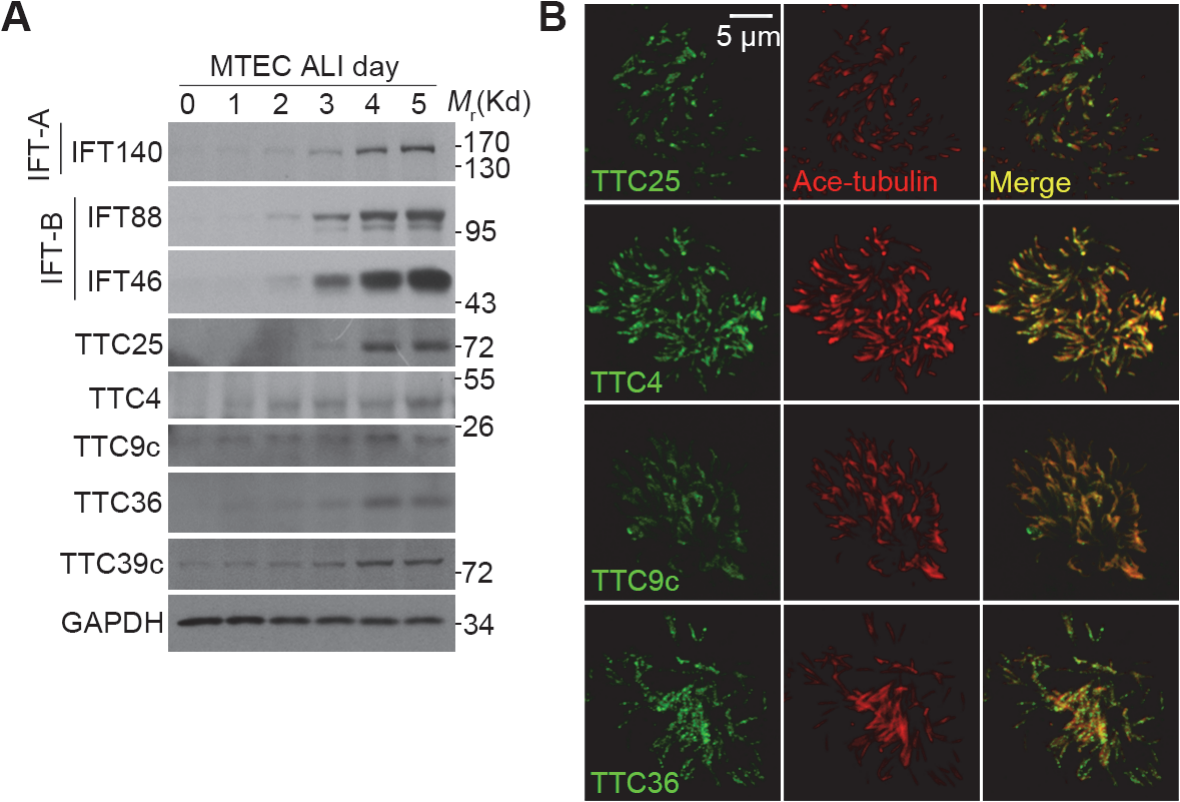
Localizations of TTC4, -9c, -25, and -36 in the multicilia of MTECs. (A) The expression profiles of the indicated proteins during MTEC differentiation into multiciliated cells. GAPDH was used as loading control. (B) The cilia localizations of the indicated proteins in MTECs at ALI d7. Acetylated tubulin was used to mark the ciliary axonemes.

All these TTC genes are conserved in the evolution and their human proteins shared more than 50% sequence identities with their zebrafish homologs (S4 Fig). To further study functions of TTC25 and the four novel TTC proteins in mammals, we generated their polyclonal antibodies. Consistent with the expression profiles (Fig 1C and 1D), the levels of all these TTC proteins increased following ALI (Fig 5A). GFP-tagged *Xenopus* TTC25 is known to locate at both cilia base and axonemes [22]. Similar localizations were observed for endogenous TTC25 in multiciliated MTECs (Fig 5B). TTC4, TTC9c, and TTC36 were also markedly enriched in the ciliary axonemes in the MTECs (Fig 5B). Antigen competition experiments also confirmed that the immunofluorescent signals were specific (S5 Fig). As to TTC39c, we were not sure of its subcellular localization because the antibody did not work for immunostaining.

### The TTC proteins associate with components of IFT complex or BBSome

**Fig 6:**
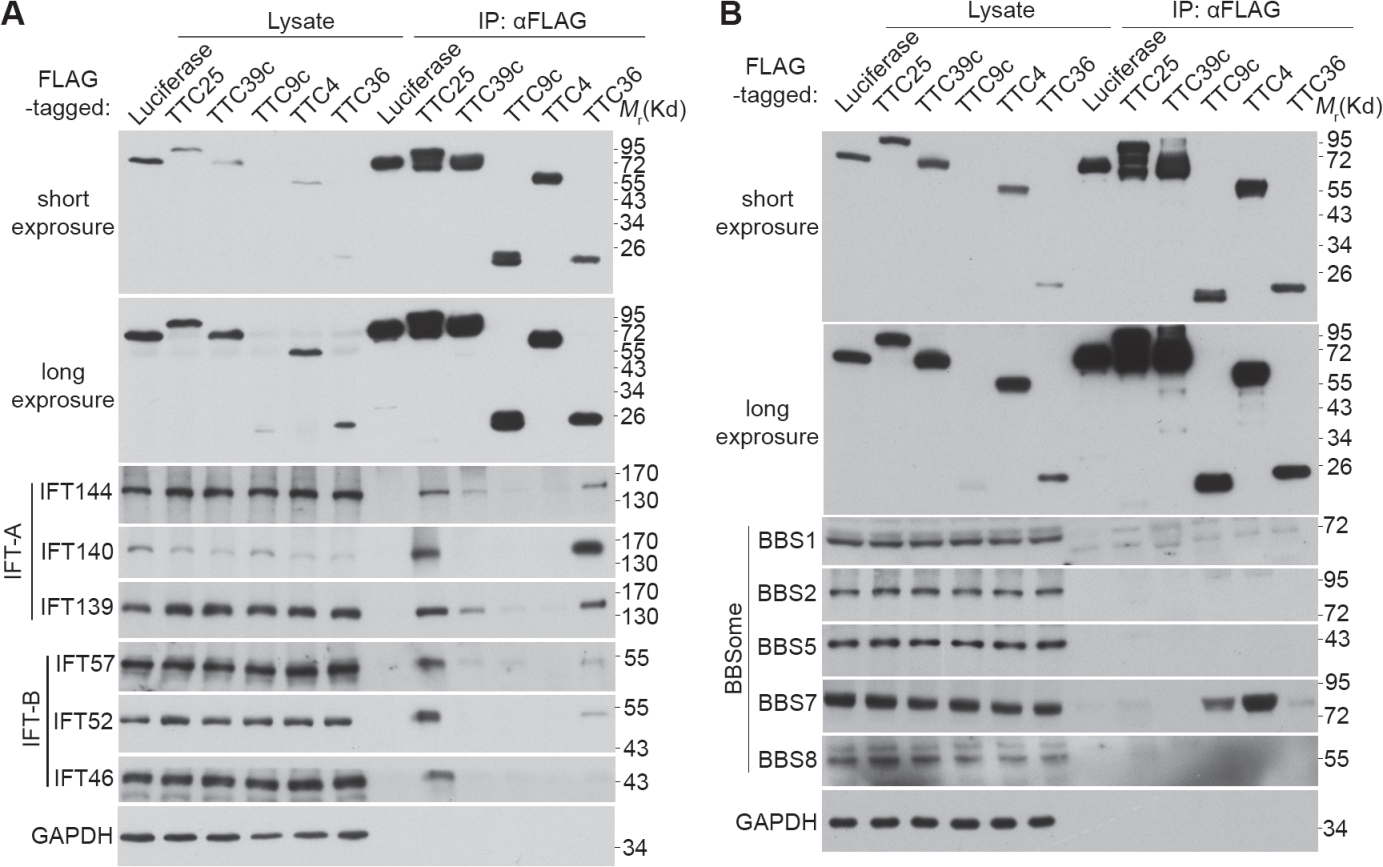
Associations of the indicated TTC proteins with IFT complexes and BBSome. HEK293T cell lysates containing the indicated FLAG-tagged TTC proteins were mixed respectively with mouse testis lysates and subjected to co-immunoprecipitation with anti-FLAG M2 resin. Flag-luciferase was used as negative control. Immunoblotting was then performed to detect the FLAG-tagged proteins and representative components of the IFT complexes (A) or BBSome (B).

In addition to the abundance of TTC proteins in the IFT complexes and BBSome [18–20], TTC26, its trypanosome ortholog PIFTC3, and *Chlamydomonas* ortholog DYF13 also associate with IFT-B [21,44]. Therefore, for further insights into the roles of the TTC proteins, we investigated whether they could interact with the IFT complexes and BBSome as well. We mixed HEK293T cell lysates expressing FLAG-tagged TTC proteins with mouse testis lysates, which were abundant in proteins of IFT complex and BBSome (Fig 6A and 6B). Before the coimmunoprecipitation with anti-FLAG resin, the mixtures were pre-incubated for two hours to allow for the complex formation [45]. Immunoblotting indicated that TTC25 exhibited strong interaction with the tested components of both IFT-A and IFT-B complexes, suggesting an association with both complexes (Fig 6A). TTC36 mainly interacted with IFT-A complex, whereas TTC39c showed weak association with IFT144 and IFT139, two subunits of IFT-A. By contrast, although TTC4 and TTC9c hardly associated with the IFT complexes (Fig 6A), they interacted specifically with BBS7 (Fig 6B). Reciprocal GST-pull down experiments further confirmed that the interactions between BBS7 and TTC4 or TTC9c were direct (S6 Fig).

## Discussion

Our study provides a global view for the relationship between TTC genes and cilia functions. Proteomic analysis, cDNA microarray, and high throughput *in situ* hybridization have previously been used to identify cilia-related proteins or genes from isolated cilia, MTECs, or *Xenopus* or zebrafish embryos [22,46–49]. FoxJ1, the master transcription factor of motile cilia [50,51], is shown to drive the expression of several TTC genes, including *IFT88*, *IFT140*, *IFT172*, and *TTC26* [48], whereas TTC25 is found critical for multicilia formation in *Xenopus* embryonic skin cells [22]. We performed cDNA microarray using DAPT-treated MTECs and found that, in addition to ten known cilia-related TTC genes, ten novel TTC genes also exhibited correlated expression patterns during multiciliogenesis (Fig 1). Consistently, all the ten novel TTC genes, except *TTC5 and TTC39c*, have been found to be upregulated by at least two-fold in the previous cDNA microarray using FoxJ1-positive MTECs [46]. We characterized these ten novel genes using zebrafish embryos and found that *ttc4*, *-9c*, *-36*, and *-39c* are critical for motile cilia functions. The remaining six, *ttc5*, *-12*, -*16*, *-18*, *-29*, and *-39a*, were precluded from our further studies because their zebrafish morphants did not show striking body curvatures (Fig 2). Nevertheless, since we did not check the knockdown efficiency of the MOs, whether these genes are indeed unrelated to cilia still needs further clarification.

Our results indicate that, TTC4, -9c, -36, -39c, and -25 are critical for normal body shape, otolith formation, pronephric cilia function, and left-right asymmetry during zebrafish embryonic development (Figs 2, 3 and 4). They are also required for proper fluid flow of the brain ventricles (Fig 3) [32]. Kidney cysts are mainly attributed to overproliferation [52–54]. In zebrafish, defects in motile cilia can cause pronephric cysts but detailed mechanisms are still poorly understood [32,52,54]. Although the *ttc* morphants frequently exhibited enlarged pronephric ducts (S2-S6 Videa vs. S1 Video), only some morphants of *ttc4* and *ttc25* contained visible pronephric cysts (Fig 3). Nonetheless, there might exist tiny cysts that were not visible in our examinations. Detailed studies in the future are still required to clarify whether or not *ttc9c*, *ttc36*, and *ttc39c* are involved in pronephric cyst formation.

Although all these TTC proteins are important for KV cilia formation (Fig 4A–4C), they have different impact on the motility of pronephric cilia (Fig 4E and 4F and S2, S3, S4, S5 and S6 Videos). Compared to the control morphants, pronephric cilia motilities were strikingly repressed in the morphants of *ttc9c*, *ttc36*, and *ttc39c*. The average CBF was reduced by more than 81% (Fig 4E and 4F). By contrast, in the morphants of *ttc25* and *ttc4*, the reduction in average CBF was less than 40% (Fig 4E and 4F). This suggests that *ttc9c*, *ttc36*, and *ttc39c* may have a role in the assembly or activity of axonemal dynein arms.

It is both surprising and interesting to find that all the cilia-related TTC proteins are implicated in IFT. Nine TTC proteins, including TTC26, have previously been shown as components of IFT-A or-B complex or BBSome [18–21] (Fig 1B). TTC25 is important for ciliogenesis and hedgehog signaling in *Xenopus* [22]. We further showed that mammalian TTC25 is strongly associated with both IFT-A and—B complexes but not BBSome (Fig 6). Therefore, TTC25 might function in bridging IFT-A and IFT-B during IFT. TTC36 appeared to associate more strongly with IFT-A complex than with IFT-B complex, whereas TTC39c, -4, and -9c associated with certain subcomplex or subunits of IFT-A or BBSome (Fig 6). Since TTC26/DYF13 is involved in the transport of motility-related proteins into cilia/flagella through association with IFT-B [21], the association of TTC36 with IFT-A might also function in transport of certain cilia components. TTC36 (also named HBP21) and TTC4 have been proposed as co-chaperons implicated in tumorigenesis [55,56] and might help protein folding as well. The punctate staining patterns of these TTC proteins (Fig 5B) are also consistent with their potential roles in IFT. Nevertheless, how these novel cilia-related TTC proteins function at the molecular level still needs future investigations.

## Supporting Information

S1 Fig Dose-dependent effects of the *ttc* MOs. One-cell-stage zebrafish embryos were injected with the indicated dose of the MOs. The images were taken at 72 hpf.
(TIF)

S2 Fig qPCR results of the indicated genes during MTEC differentiation into multiciliated cells. One of the two biological replicates is shown here. mRNA levels at ALI day 0 were set at 1 for normalization. Another set of data is depicted in Fig 1D.
(TIF)

S3 Fig GFP reporter assays for MO efficiency. The indicated *ttc*-GFP reporter mRNAs were coinjected with the corresponding MOs or *ctrl*-MO into zebrafish embryos. The brightfield and fluorescent images were taken at 12 hpf. The expression of GFP was suppressed by the *ttc* MOs but not *ctrl*-MO.
(TIF)

S4 Fig Schematic comparison between human and zebrafish TTC homologs. The protein sequences used for similarity analyses were from GenBank accession numbers NP_113609 (Human TTC25), NP_956610 (Zebrafish TTC25), NP_004614 (Human TTC4), NP_001002122 (Zebrafish TTC4), NP_776171 (Human TTC9c), NP_956559 (Zebrafish TTC9c), NP_001073910 (Human TTC36), NP_001007389 (Zebrafish TTC36), NP_001129465 (Human TTC39c), NP_001018404 (Zebrafish TTC39c). Orange boxes indicate TPR motifs.
(TIF)

S5 Fig Validation of TTC antibody specificity. Antibodies against the indicated TTC proteins were pre-incubated with either GST (A) or GST-tagged antigens (B) for 2 h and then used for immunostaining of multiciliated MTECs. Acetylated tubulin was used to mark the ciliary axonemes.
(TIF)

S6 Fig TTC4 and TTC9c directly interact with BBS7. Bacterial lysates containing the indicated His-tagged TTC proteins were mixed with GST-tagged BBS7 and subjected to GST pull-down assays. His-tagged luciferase was used as negative control.
(TIF)

S7 Fig Full scans of original blots. The boxed regions indicate the blots shown in the figures.
(TIF)

S1 Table The sequences of primers used. (XLSX)

S2 Table Expression profile of selected genes based on cDNA microarray analysis. (XLSX)

S1 Video Pronephric cilia motilities in a typical control zebrafish embryo at 60 hpf. The video was captured at 200 frames per sec (fps) and replayed at 30 fps. Anterior is to the left.
(MOV)

S2 Video Pronephric cilia motilities in a typical *ttc25* morphant at 60 hpf. The video was captured at 200 fps and replayed at 30 fps. Anterior is to the left.
(MOV)

S3 Video Pronephric cilia motilities in a typical *ttc4* morphant at 60 hpf. The video was captured at 200 fps and replayed at 30 fps. Anterior is to the left.
(MOV)

S4 Video Pronephric cilia motilities in a typical *ttc9c* morphant at 60 hpf. The video was captured at 200 fps and replayed at 30 fps. Anterior is to the left.
(MOV)

S5 Video Pronephric cilia motilities in a typical *ttc36* morphant at 60 hpf. The video was captured at 200 fps and replayed at 30 fps. Anterior is to the left.
(MOV)

S6 Video Pronephric cilia motilities in a typical *ttc39c* morphant at 60 hpf. The video was captured at 200 fps and replayed at 30 fps. Anterior is to the left.
(MOV)
