# Supplementary figures and images for: Characterization of Tetratricopeptide Repeat-Containing Proteins Critical for Cilia Formation and Function
Source: PLoS One. 2015 Apr 10;10(4):e0124378. doi: 10.1371/journal.pone.0124378 (PMC4393279; doi:10.1371/journal.pone.0124378)

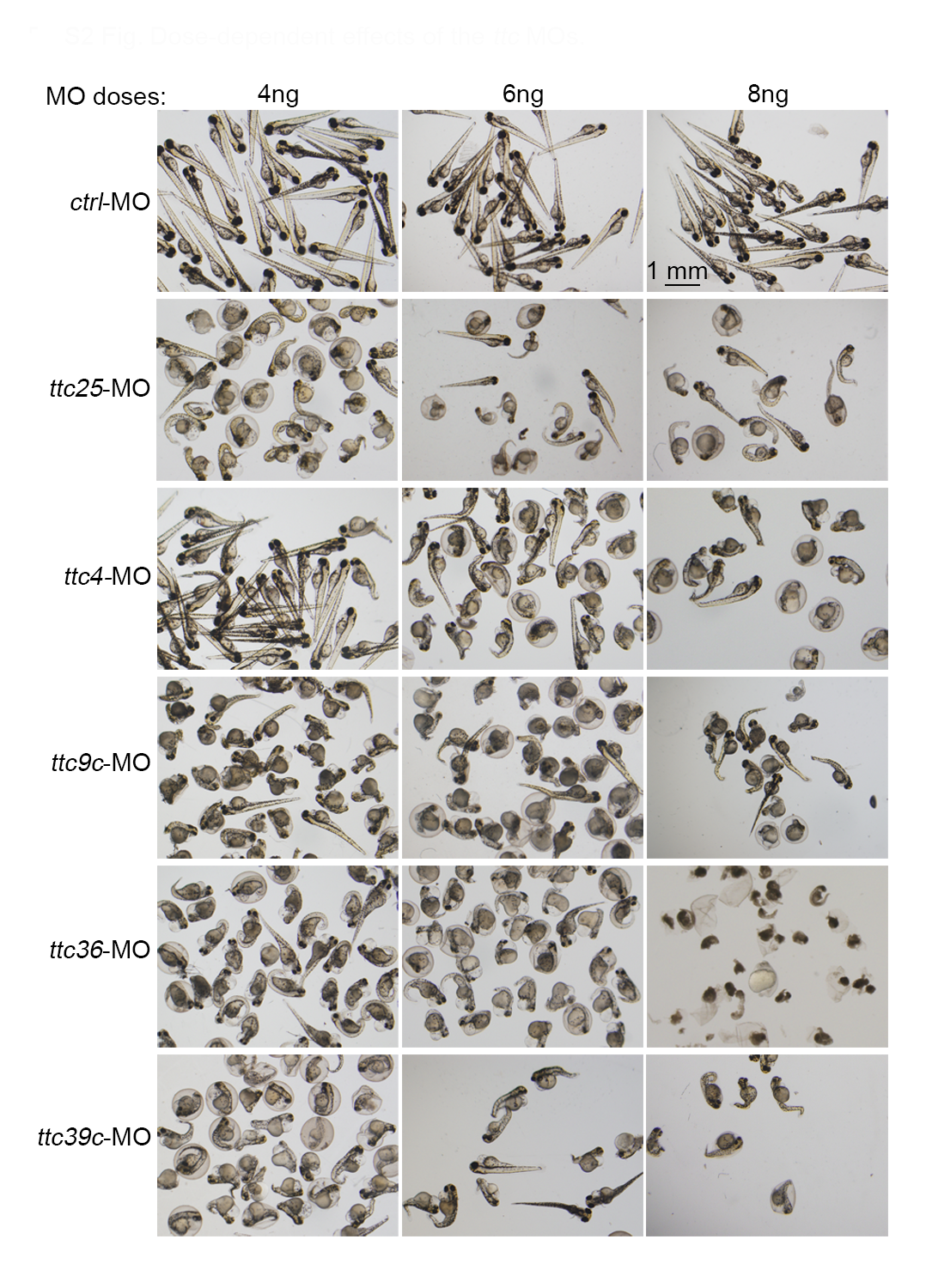

Supplement: S1 Fig — One-cell-stage zebrafish embryos were injected with the indicated dose of the MOs. The images were taken at 72 hpf. (TIF) [file pone.0124378.s001.tif]

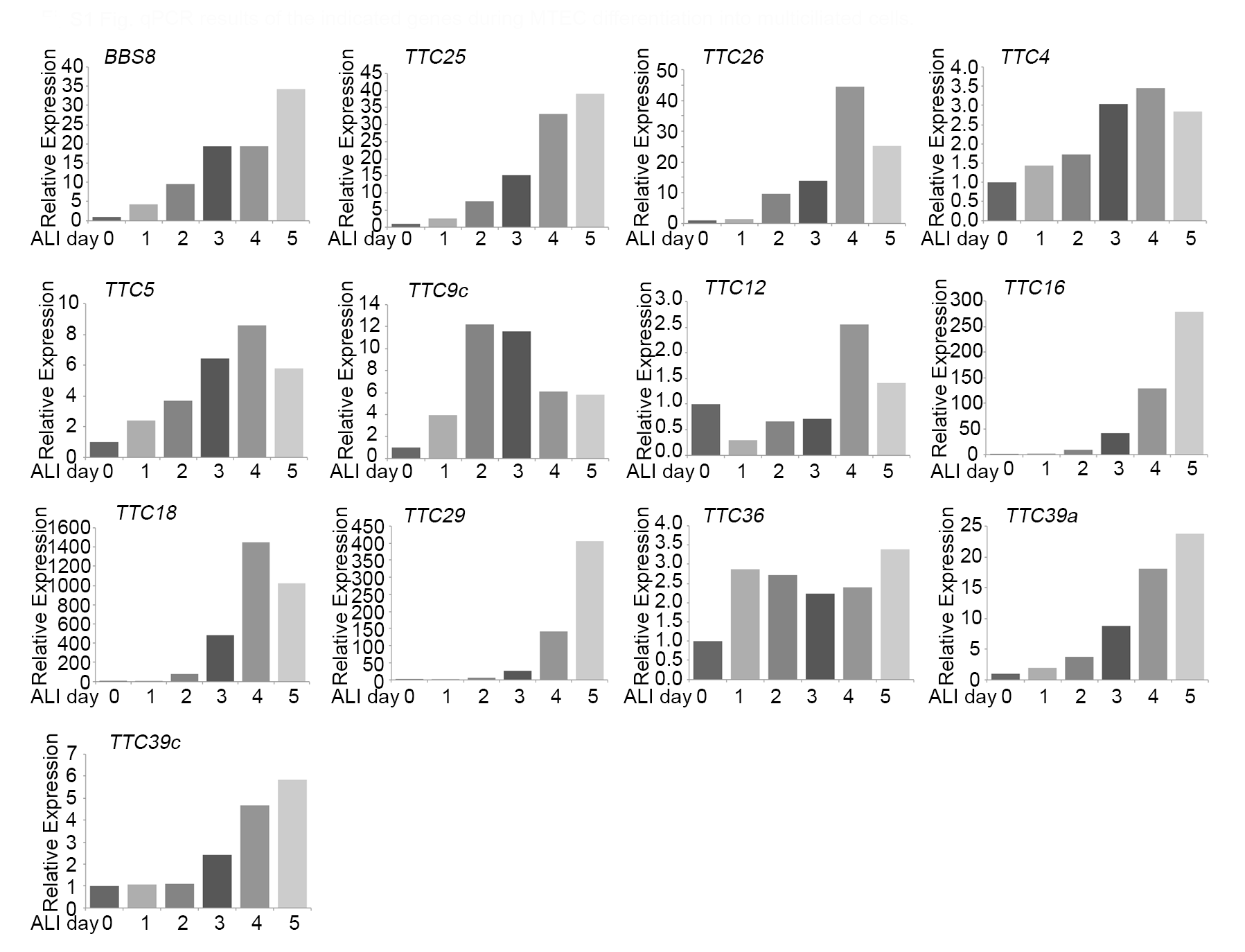

Supplement: S2 Fig — One of the two biological replicates is shown here. mRNA levels at ALI day 0 were set at 1 for normalization. Another set of data is depicted in Fig 1D. (TIF) [file pone.0124378.s002.tif]

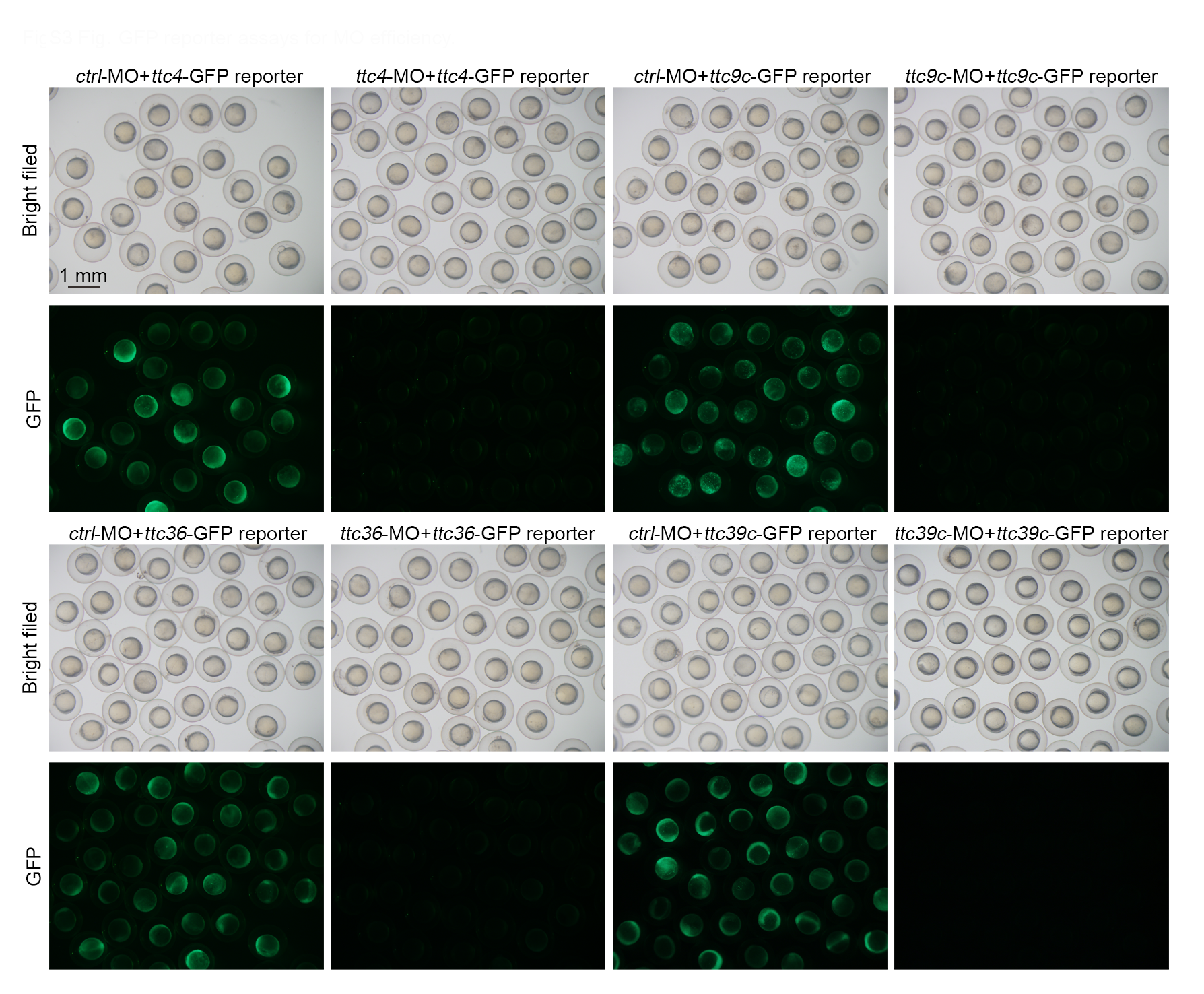

Supplement: S3 Fig — The indicated ttc-GFP reporter mRNAs were coinjected with the corresponding MOs or ctrl-MO into zebrafish embryos. The brightfield and fluorescent images were taken at 12 hpf. The expression of GFP was suppressed by the ttc MOs but not ctrl-MO. (TIF) [file pone.0124378.s003.tif]

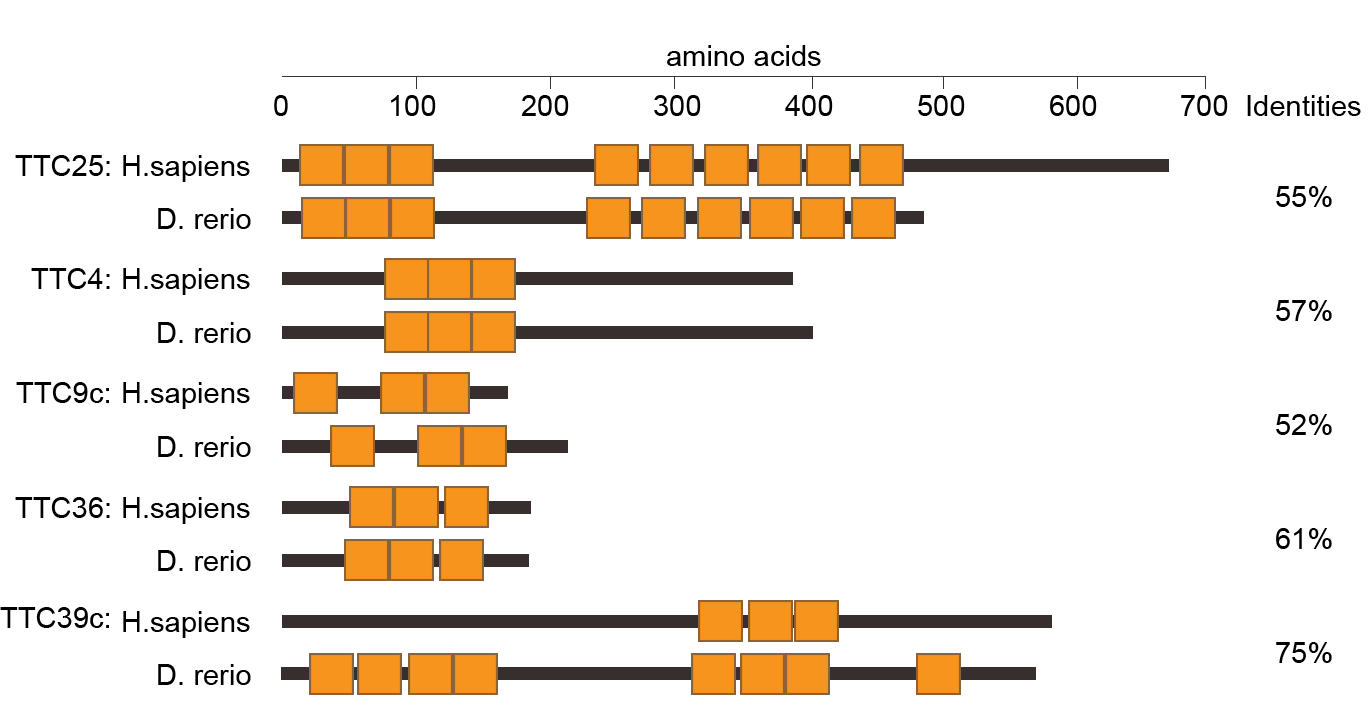

Supplement: S4 Fig — The protein sequences used for similarity analyses were from GenBank accession numbers NP_113609 (Human TTC25), NP_956610 (Zebrafish TTC25), NP_004614 (Human TTC4), NP_001002122 (Zebrafish TTC4), NP_776171 (Human TTC9c), NP_956559 (Zebrafish TTC9c), NP_001073910 (Human TTC36), NP_001007389 (Zebrafish TTC36), NP_001129465 (Human TTC39c), NP_001018404 (Zebrafish TTC39c). Orange boxes indicate TPR motifs. (TIF) [file pone.0124378.s004.tif]

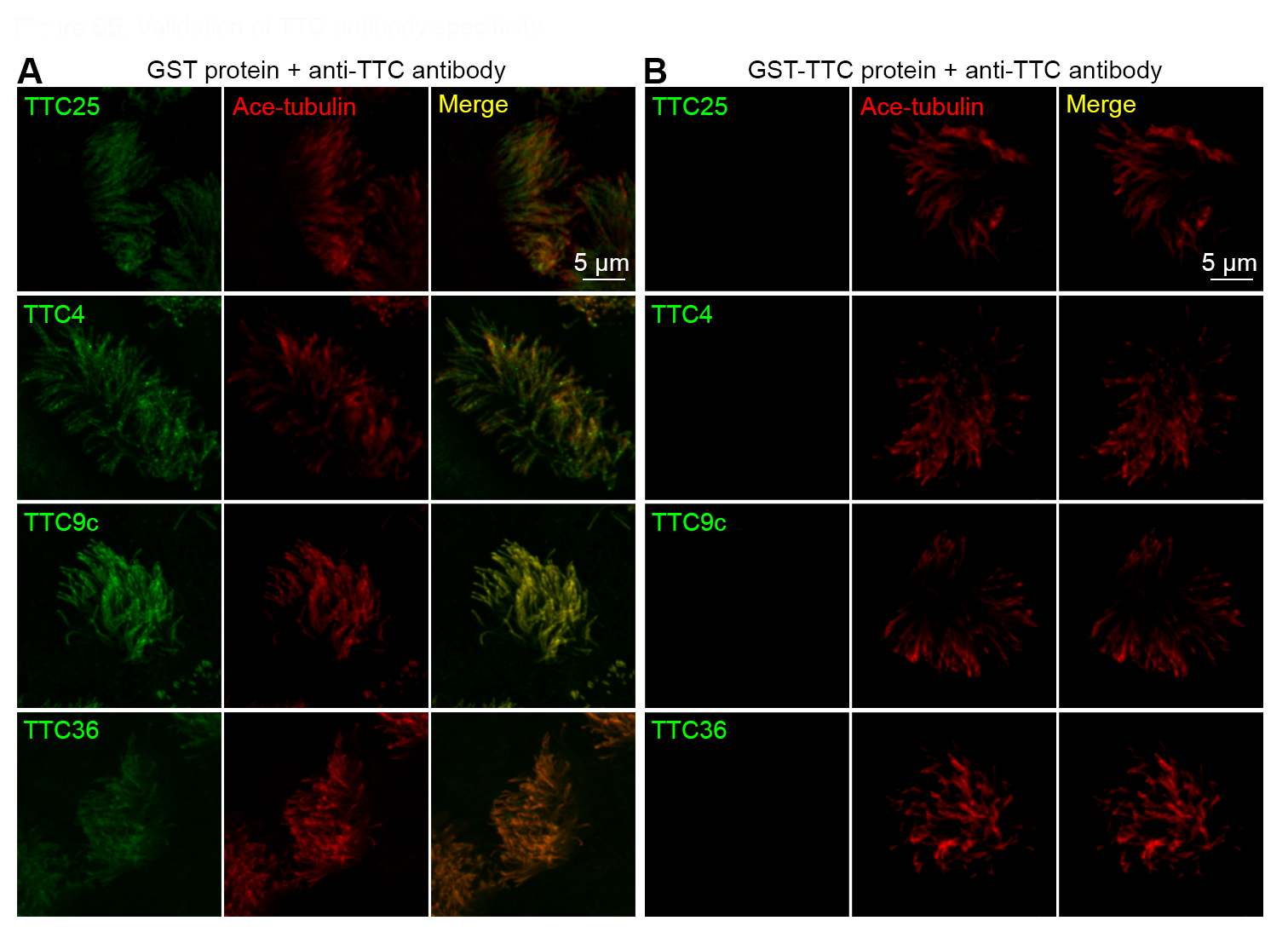

Supplement: S5 Fig — Antibodies against the indicated TTC proteins were pre-incubated with either GST (A) or GST-tagged antigens (B) for 2 h and then used for immunostaining of multiciliated MTECs. Acetylated tubulin was used to mark the ciliary axonemes. (TIF) [file pone.0124378.s005.tif]

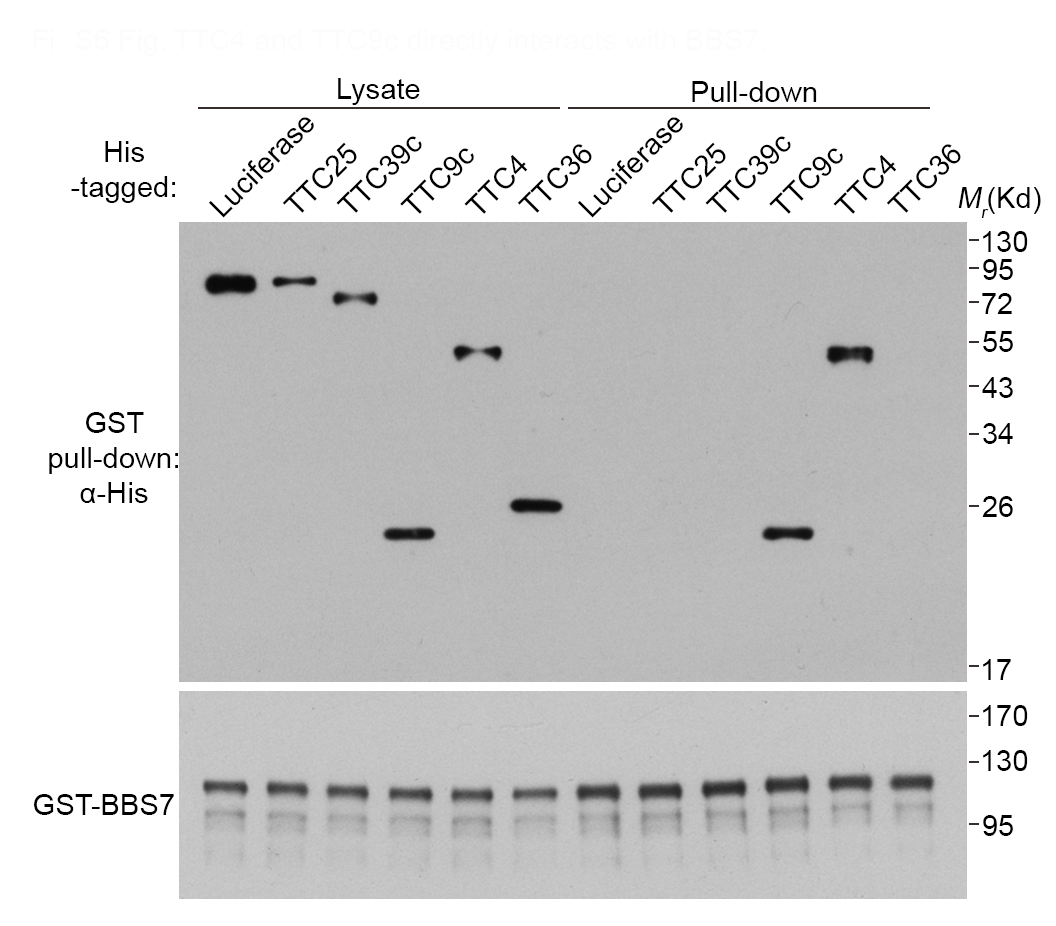

Supplement: S6 Fig — Bacterial lysates containing the indicated His-tagged TTC proteins were mixed with GST-tagged BBS7 and subjected to GST pull-down assays. His-tagged luciferase was used as negative control. (TIF) [file pone.0124378.s006.tif]

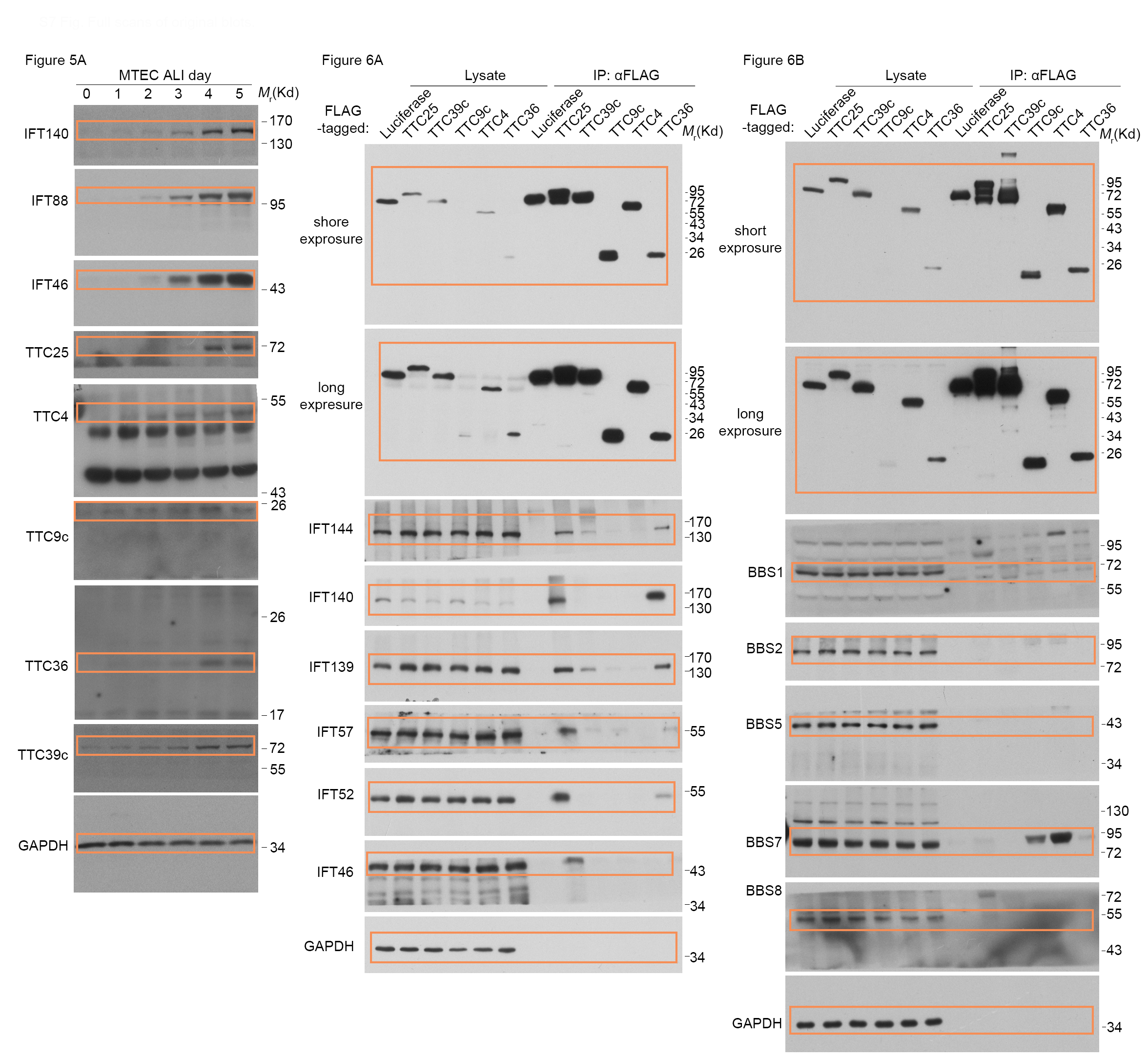

Supplement: S7 Fig — The boxed regions indicate the blots shown in the figures. (TIF) [file pone.0124378.s007.tif]
